# A Deeper Depth of Response After Salvage Therapy Improves Outcomes of Autologous Stem Cell Transplantation in Relapsed Lymphoma and the Feasibility of Non-controlled Rate Freezing of Peripheral Blood Stem Cells

**DOI:** 10.7759/cureus.56851

**Published:** 2024-03-24

**Authors:** Saif ur Rab, Mussadique Ali, Uzma Rasool Mahar, Bushra Ahsan, Usman Ahmad, Muhammad Tariq Mahmood, Neelam Siddiqui, Syed W Bokhari

**Affiliations:** 1 Medical Oncology, Shaukat Khanum Memorial Cancer Hospital and Research Centre, Lahore, PAK; 2 Medical Oncology-Bone Marrow Transplant, Shaukat Khanum Memorial Cancer Hospital and Research Centre, Lahore, PAK; 3 Pathology, Shaukat Khanum Memorial Cancer Hospital and Research Centre, Lahore, PAK

**Keywords:** peripheral blood stem cells, freezing, response, lymphoma, autologous transplant, salvage chemotherapy

## Abstract

Background

High-dose chemotherapy followed by autologous stem cell transplantation is considered a standard treatment approach for patients with relapsed Hodgkin's lymphoma (HL) and non-Hodgkin lymphoma (NHL). The goal of autologous stem cell transplant in relapsed lymphoma is to achieve long-term disease control, i.e., cure, in contrast to disorders like multiple myeloma, where it only prolongs the duration of remission, progression-free survival, and improves the quality of life. Published outcomes of high-dose therapy and ASCT and the impact of different factors affecting survival in low- to middle-income countries are very limited. Our study analyzed all the autologous stem cell transplants performed in our center over a six-year period to ascertain engraftment, responses, outcomes, and variables that may have impacted transplant outcomes.

Methods

We conducted a retrospective study including 76 patients from January 2015 to December 2020. Data were retrieved from electronic medical records at Shaukat Khanum Memorial Cancer Hospital and Research Centre, Lahore, Pakistan.

Results

Out of a total of 82 autologous transplant patients, 76 were eligible for the study, out of which 50 (66%) had HL and 26 (34%) had NHL. The median age was 29 years (range 18-53) and 29 years (range 20-45) for HL and NHL, respectively. The male-to-female ratio was 5:2 and 4:1 for HL and NHL, respectively. The majority had advanced-stage disease, 85% in HL and 75% in NHL. The minimum cell dose infused was 2.5 million CD34+ cells/kg. Median days to platelets and ANC engraftment were 14 and 11 days, respectively. The 30-day transplant-related mortality was 8.9% and 7.4% in HL and NHL, respectively. The 100-day mortality was 15.2% and 11% in HL and NHL, respectively. The two-year disease-free survival (DFS) and overall survival (OS) were 83% and 83%, respectively, in HL patients. The two-year DFS and OS were 78% and 85%, respectively, in NHL patients.

Conclusion

High-dose therapy and autologous stem cell transplantation in low- to middle-income countries are limited to relatively younger patients, potentially curative conditions such as lymphoma, and predominantly after achieving a complete response to salvage therapy due to limited resources. Due to these factors, our study shows excellent response rates and survival outcomes compared to internationally published data. Engraftment was also excellent and comparable to published data despite the non-controlled rate freezing of peripheral blood stem cells.

## Introduction

High-dose chemotherapy and autologous stem cell transplant (ASCT) are considered standard treatment approaches for patients with relapsed Hodgkin's lymphoma (HL), relapsed non-Hodgkin's lymphoma (NHL), multiple myeloma (MM), and a few solid cancers such as mixed germ cell tumors. The goal of ASCT in relapsed lymphoma is to achieve long-term disease control, i.e., cure, in contrast to MM, where it achieves prolongation of the duration of remission, progression-free survival, and improvement in quality of life.

The annual activity survey of the European Society for Blood and Marrow Transplantation (EBMT) 2019 showed that 58.6% of all transplants were ASCT and 41% were allogeneic stem cell transplants. Most of these ASCTs were done for plasma cell disorders (46%) followed by NHL (30%) and HL (11%)[1]. The shift from bone marrow to peripheral blood as a source of stem cells occurred in the early 1990s for ASCT. Recent data now suggest that 99% of all ASCT procedures use peripheral blood as the source of stem cells [2]. For this purpose, peripheral blood stem cells are mobilized using G-CSF in combination with chemotherapy or plerixafor, and collected cells are cryopreserved. Although it is preferred to cryopreserve stem cells in a controlled-rate freezer with liquid or vapor-phase nitrogen at -180°C [3-5], not many centers in low- to middle-income countries, including ours, have developed this facility due to its cost, and cells are routinely stored in mechanical freezers at -80°C with uncontrolled rate direct freezing. Some studies have shown this method of freezing to be safe although there is still concern about damage to the stem cell population due to heat liberation with uncontrolled direct freezing [6,7]. Its safety and impact on engraftment need to be ascertained in a large number of patients.

The standard management of relapsed/refractory HL and NHL is with intensive salvage chemotherapy followed by high-dose chemotherapy with ASCT in patients with a partial response (PR) or better. This results in long-term disease-free survival of 40-60% [8,9]. Although several factors are considered important, chemosensitivity with at least a partial response to salvage therapy is considered a requirement to proceed to ASCT [10]. There is some role of ASCT shown in chemosensitive primary refractory HL as well [11]. Retrospective studies suggest superior outcomes with ASCT if patients achieve complete remission (CR) before ASCT [12-14]. ASCT remains a key component of MM therapy in eligible patients as well and is mostly incorporated as part of the initial therapy.

Published outcomes of high-dose therapy and ASCT and the impact of different factors affecting survival in low- to middle-income countries are very limited. Our study analyzed all the autologous stem cell transplants performed in our center to ascertain responses, outcomes, and variables that may have impacted transplant outcomes.

This is a retrospective study of relapsed and/or refractory HL and NHL patients who have received ASCT at Shaukat Khanum Memorial Cancer Hospital and Research Centre, Lahore, Pakistan. Assessment of response rates and post-ASCT outcomes (post-ASCT engraftment, transplant-related mortality, DFS, OS) and the impact of pre-ASCT variables, particularly depth of response pre-ASCT by CT and/or PET, amongst other factors on outcomes (DFS, OS), were analyzed.

## Materials and methods

Objectives 

The primary aim of this study was to assess response rates, post-ASCT engraftment, TRM, and outcomes in terms of DFS and OS in HL and NHL patients treated with salvage chemotherapy followed by ASCT. The secondary aim was to assess the effect of various factors on DFS and OS including the stage at diagnosis, B symptoms, baseline risk score, and depth and duration of response to first-line at the time of transplant. 

Methods 

This was a retrospective study that included all consecutive patients with HL and NHL who underwent ASCT at our institute during the period from January 2015 to December 2020. 

Inclusion Criteria

The inclusion criteria include all patients >18 years of age with HL and NHL who underwent ASCT between January 2015 and December 2020 at Shaukat Khanum Memorial Cancer Hospital. 

*Exclusion Criteria* 

Patients with incomplete minimal essential data and those who underwent ASCT for myeloma and solid cancer were excluded from the analysis. 

Data collection 

Data were collected from the hospital information system (HIS) with a keyword search for Hodgkin’s lymphoma, non-Hodgkin lymphoma, and autologous stem cell transplant. Data collected included age, gender, ECOG-PS, B symptoms, IPI/IPS, stage of disease at the time of the start of first-line chemotherapy, upfront treatment regimen, anti-CD20 treatment, salvage chemotherapy, autologous HCT, responses and their duration, number of cycles received, stem cell dose, day to platelet engraftment, day to neutrophil engraftment, D100 response, relapse or progression, and date and cause of death. Minimum essential data included response to salvage chemotherapy, date of autologous transplant, and response to autologous transplant assessed by day 100 PET scan, survival at D30 and D100 post-transplant, and date and cause of mortality. 

Data analysis 

The data were tabulated and analyzed using IBM SPSS Statistics for Windows, Version 21 (Released 2012; IBM Corp., Armonk, New York). Age was described as median and range; frequency was tabulated as a percentage for gender, ECOG-PS, stage, IPI/IPS, chemotherapy regimen, treatment response, as well as the duration of response at baseline, at first relapse, response, and mortality after autologous stem cell transplant. Treatment responses were documented according to standard lymphoma response criteria as complete response (CR), partial response (PR), stable disease (SD), or progressive disease (PD). OS and PFS were calculated from the point of autologous stem cell transplant, while responses are presented as percentages. Kaplan-Meier curves were used to compare survivals over time for the overall population. Further subgroup analysis was carried out with stage at diagnosis, early versus advanced stage, primary IPI and IPS (at diagnosis), response to first-line chemotherapy, and stage at second relapse, and p values <0.05 were considered statistically significant. Patients were included in the study after obtaining an exemption from the Institutional Review Board of Shaukat Khanum Memorial Trust.

## Results

Baseline characteristics

A total of 82 patients had autologous stem cell transplants between January 1, 2015, and December 31, 2020, of which data from 76 patients was gathered from the hospital information system based on inclusion and exclusion criteria. Baseline characteristics of the patients are tabulated in Table 1. Patients had an age range of 18-53 years for HL and 20-45 years for NHL. The median age was 29 years for HL and NHL with a male-to-female ratio of 5:2 and 4:1 for HL and NHL, respectively. HL patients were 66% (n=50) and NHL patients were 34% (n=26). Patients with HL had an early-stage (I and II) disease in 14% and an advanced-stage disease in 85.7%. Patients with NHL had early stage (stage I/II) in 22.5% and advanced stage (3/4) in 77.4%. 

**Table 1:** Baseline characteristics and overall and disease-free survival of Hodgkin’s and non-Hodgkin’s lymphoma patients. IPI, International Prognostic Index Score; IPS, International Prognostic Score; DFS, disease-free survival; OS, overall survival.

Characteristics	Hodgkin’s lymphoma	Non-Hodgkin’s lymphoma
Age range (years)	18-53	20-45
Median age (years)	29	29
Male-to-female ratio	5:2	4:1
Total number	50	26
Early-stage (I and II) disease	14%	22.5%
Advanced-stage (III and IV) disease	85.7%	77.4%
B Symptoms	85.7%	77.4%
Complete response to first-line treatment	40.4%	22.5%
IPS 0-3	69%	NA
IPS >4	30.9%	NA
IPI 0-3	NA	87%
IPI >4	NA	12.9%
30-day mortality	8.9%	7.4%
100-day mortality	15.2%	11%
DFS at 2 years	83%	78%
OS at 2 years	83%	85%

D30 and D100 transplant-related mortality 

The 30-day transplant-related mortality was 8.9% in HL and 7.4% in NHL. The 100-day transplant-related mortality was 15.2% in HL and 11% in NHL. 

Post-transplant engraftment 

All patients had stem cells cryopreserved and frozen by a non-controlled rate of direct freezing at -80 °C. In all patients, stem cells were cryopreserved and used within <12 months from collection. In all patients, stem cells infused were >2.5 x10^6 ^CD34+ cells/kg. The median time to platelet engraftment was 14 days (8-50) and the median time to neutrophil engraftment was 11 days (8-30). Delayed or poor engraftment was seen in four patients (5.2%). 

Post-transplant outcomes 

**Figure 1:**
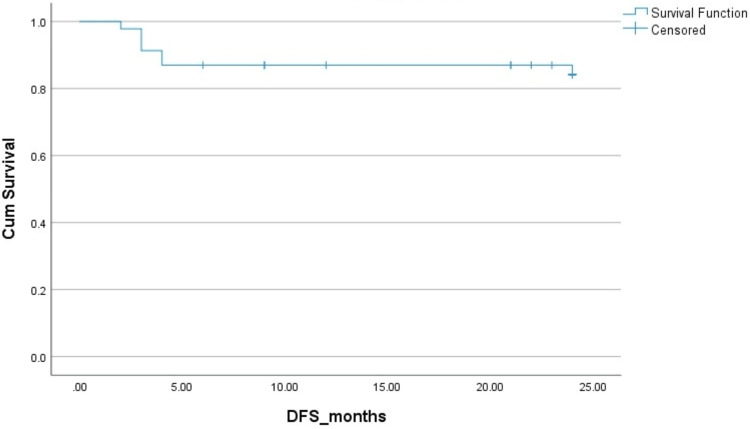
Disease-free survival (DFS) in Hodgkin's lymphoma. Cum: cumulative.

**Figure 2:**
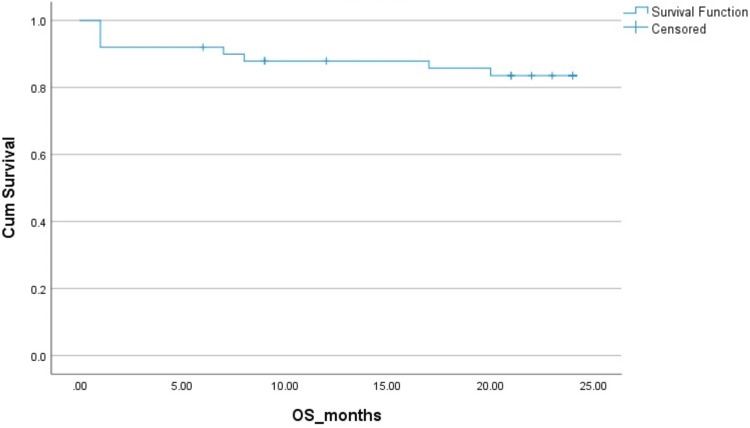
Overall survival (OS) in Hodgkin's lymphoma (HL). Cum: cumulative.

**Figure 3:**
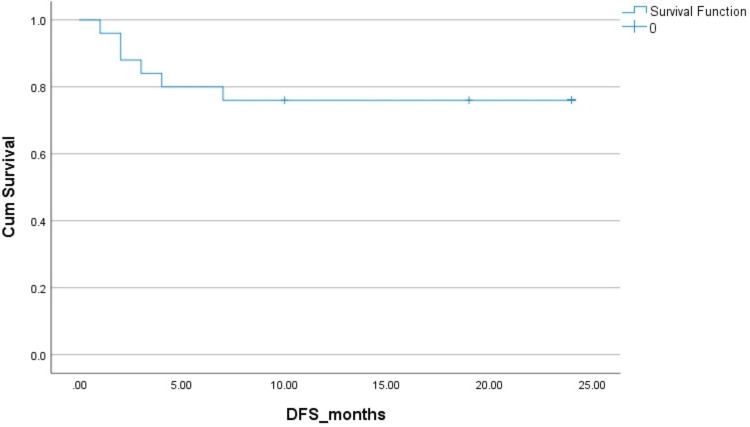
Disease-free survival (DFS) in non-Hodgkins lymphoma (NHL). Cum: cumulative.

**Figure 4:**
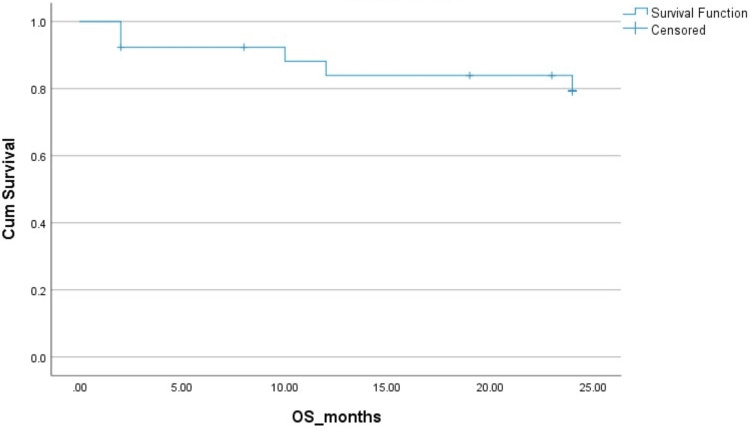
Overall survival (OS) in non-Hodgkins lymphoma (NHL). Cum: cumulative.

The two-year PFS and OS in HL patients were 83% and 83%, respectively. The two-year PFS and OS in NHL patients were 78% ​and 85%, respectively. The Kaplan-Mayer survival curves in terms of PFS and OS for HL and NHL are shown in Figures 1-4. 

Univariate analysis

Factors analyzed in the univariate analysis included the presence or absence of B symptoms, stage of disease (early versus advanced), presence or absence of extranodal sites, IPS 0-3 versus > 4 for HL, and IPI 0-3 versus > 4 for NHL, disease status at transplant, i.e., CR versus PR, and the duration of response to first-line therapy, i.e., > 12 months versus < 12 months.

In HL patients, IPS low had significantly better two-year OS compared to IPS high (p = 0.05). The presence of B symptoms and involvement of extranodal sites showed a trend towards worse OS with p values of 0.08 and 0.07, respectively.

**Figure 5:**
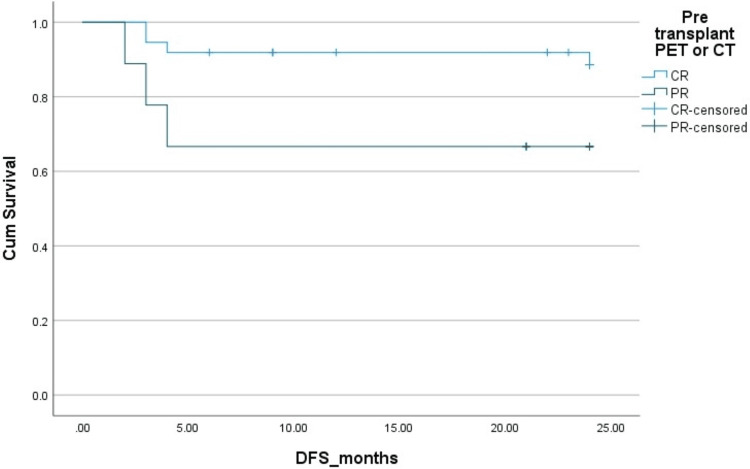
Effect of Pre-transplant disease status by PET scan on disease-free survival (DFS) in Hodgkin's lymphoma (HL). Cum, cumulative; PET, positron emission tomography; PR, partial remission; CR, complete remission.

**Figure 6:**
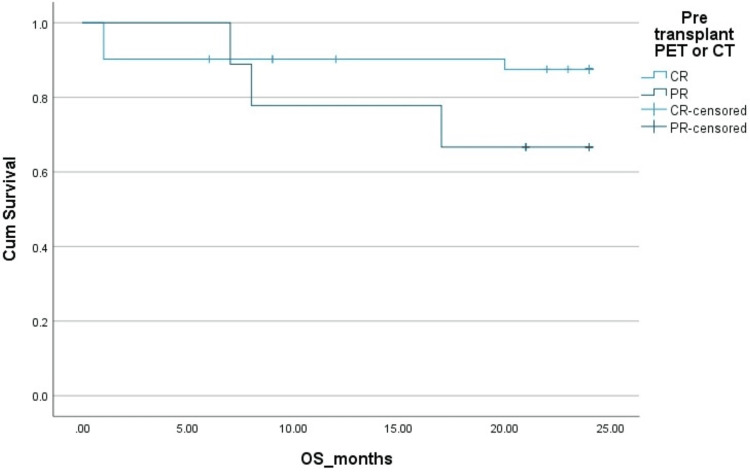
Effect of pre-transplant disease status by PET scan on overall survival (OS) in Hodgkin's lymphoma (HL). Cum, cumulative; PET, positron emission tomography; PR, partial remission; CR, complete remission.

**Figure 7:**
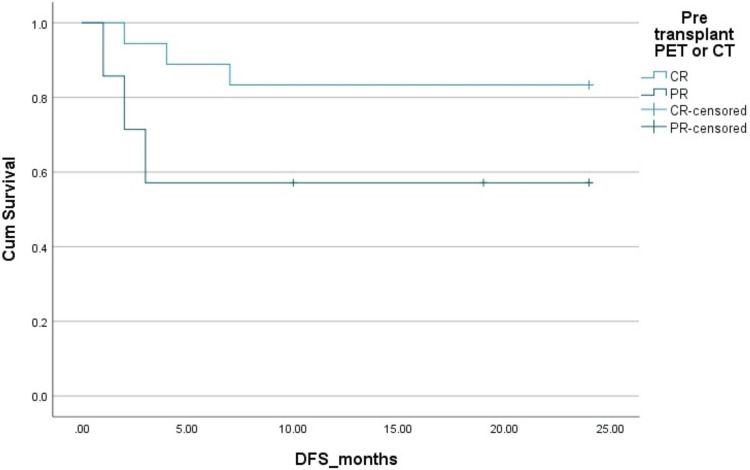
Effect of pre-transplant disease status by PET scan on disease-free survival (DFS) in non-Hodgkins lymphoma (NHL). Cum, cumulative; PET, positron emission tomography; PR, partial remission; CR, complete remission.

**Figure 8:**
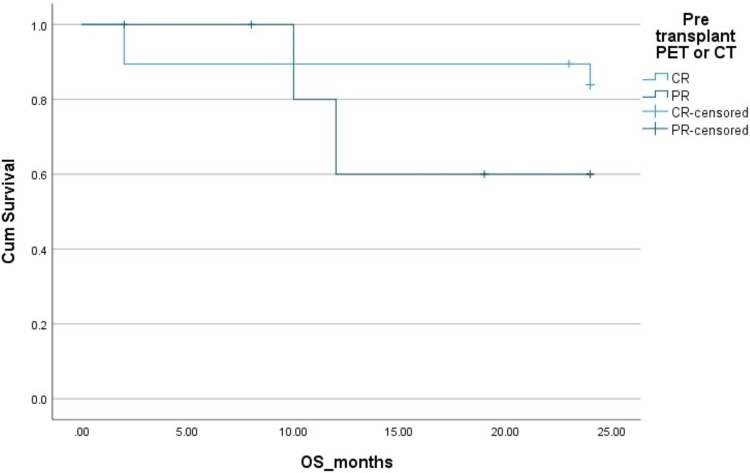
Effect of pre-transplant disease status by PET scan on overall survival(OS) in non-Hodgkins lymphoma (NHL). Cum, cumulative; PET, positron emission tomography; PR, partial remission; CR, complete remission.

In NHL patients, early stage and IPI of 0-3 had significantly better OS with p values of 0.02 and 0.02, respectively. Results are shown in Table 2 and Figures 5-8.

**Table 2:** Univariate analysis IPS: International Prognostic Score; IPI: International Prognostic Index; DFS: disease-free survival; OS: overall survival.

Characteristics	Hodgkin’s lymphoma					Non-Hodgkin’s lymphoma	
	OS	DFS	P value	OS	DFS		P value
B symptoms							
Yes	66%		0.08	87%			0.5
No	100%			100%			
Stage							
I/II	85.7%		0.1	100%			0.02
III/IV	76%			87%			
Extra-nodal site							
Yes	33%		0.07	88%			0.8
No	90%			100%			
IPI/IPS							
0-3	85%		0.05	100%			0.02
>4	65%			77%			
Pre-transplant disease status							
Complete response	88%	92%	0.1 (OS), 0.05 (DFS)	88%	85%		0.2 (OS), 0.09 (DFS)
Partial response	70%	68%		60%	58%		

## Discussion

The use of peripheral blood stem cells for autologous and allogeneic transplantation has increased significantly. The 76 patients reported in this study, who received an ASCT as a treatment for relapsed/refractory HL and NHL between January 2015 and December 2020, represent the longest reported follow-up period from a single center in a low- to middle-income country and show long-term DFS and OS. Our study indicates that in low- to middle-income countries, in contrast to the developed world, the vast majority of ASCTs are being performed for diseases such as HL and NHL with curative intent, and very few transplants have been done in diseases such as myeloma, where it is considered to be a non-curative treatment. Additionally, our study shows that the proportion of patients going into transplant with a CR was significantly higher than in PR, suggesting that perhaps fewer PR patients were accepted for ASCT.

In this study, we demonstrated that the outcomes in terms of OS and DFS of the whole group of 76 patients were in accordance with published international studies, despite being in a resource-constrained area with limited options available. In fact, two-year DFS and OS seem to be even better than published data in both HL and NHL. This could partly be due to a younger and fitter group of patients but partly due to a higher proportion of patients with CR going into transplant. The cost of an autologous transplant at our institution was approximately $20,000, and almost all transplants carried out in our data were hospital-funded. Our institute is a charitable organization with limited resources, and this has led to the selection of comparatively younger patients with deeper responses to salvage chemotherapy for autologous transplant, which is perhaps a reason behind better disease-free and overall survival.

As previously mentioned, our center, like many in low-middle-income countries, utilizes -80°C mechanical freezers to cryopreserve stem cells. To mitigate the risk of delayed engraftment or graft failure, we use cryopreserved stem cells stored for less than 12 months and have a higher minimum stem cell dose target of 2.5 million CD34+ cells/kg. Our study shows excellent engraftment figures in keeping with published data. This approach to cryopreservation and storage hence could be safely used by centers in low-middle-income countries where controlled rate freezing in liquid or vapor-phase nitrogen at -180°C is too costly, labor-intensive, and not affordable.

Previous studies have shown that second-line IPI or age-adjusted IPI at relapse can help differentiate patients with relapsed refractory NHL, who are less likely to benefit when treated with ASCT [15,16]. As a surrogate marker of disease biology, IPI at baseline has been used to predict survival in treatment-naive aggressive NHL; however, its effect on outcomes after ASCT has not been studied thoroughly. Our study shows that higher IPS and IPI in HL and NHL, respectively, are associated with inferior outcomes. Baseline IPI and IPS seem to identify patients at high risk of transplant failure even before second-line treatment is undertaken.

We recognize the limitations of the study, such as it being a single-center retrospective study, long study duration, and a relatively smaller number of patients.

## Conclusions

High-dose therapy followed by autologous stem cell transplantation in low-middle-income countries is limited to relatively younger patients, potentially curative conditions such as lymphoma, and predominantly after achieving a complete response to salvage therapy due to limited resources. Due to these factors, our study shows excellent response rates and survival outcomes compared to internationally published data. Cryopreservation and storage of stem cells in -80°C freezers without a controlled rate of freezing, re-infusion of stem cells with no longer than a maximum of 12 months of storage, and a higher minimum stem cell dose of >2.5 million CD34+ cells/kg are feasible and resulted in excellent post-transplant engraftment comparable to published data.
